# Keystone Veterinary Medical Association

**Published:** 1891-01

**Authors:** C. H. Magill

**Affiliations:** Secretary


*Keystone Veterinary Medical Association.*—The Keystone Veterinary Medical Association held its regular meeting at the College of Physicians, Philadelphia, December 6th, 1890, the president, Dr. Hoskins, in the chair. Present: Drs. Zuill, Glass, Hoskins, Huidekoper, Kooker, Williams, Eves, Harger, Ridge, Lusson, Magill, Birch, Drake and Landes. Minutes of the November meeting were read and approved. The board of trustees had failed to hold a meeting and had no report to make. Dr. Hoskins, for the legislative committee, reported that the attorneys of the association had been instructed to draft the necessary amendments to cover the deficiencies in the present act regulating the practice of veterinary medicine. Dr. Zuill, for the committee on essays, suggested for debate: I. Should the refrigerant treatment be used to combat inflammation? II. Should veterinarians furnish their own medicines? III. Should veterinary colleges give free treatment to the owners of animals in poor circumstances?

The resolution limiting the business meetings of the association to four in the year, was brought up and amended to read that the business meetings be limited to the September, December, March and June meetings. After discussion by Drs. Glass and Williams, the resolution was put to vote and lost.

Dr. Ridge presented the name of Dr. Schrieber, and Dr. Hoskins that of Dr. Sellers for membership. The essayist, Dr. Vandegrift, was absent. Dr. Ridge presented a paper on "Pneumonia." He reviewed the etiology, pathology and treatment of the disease. He dwelt on the statement made that pneumonia does not relapse. Dr. Birch reported a case of abscess following four days after a hypodermic injection of morphia, which was followed in ten days by tetanus.

Dr. Huidekoper presented an abstract of Koch's discovery of a cure for tuberculosis. He doubted if it would prove of value in veterinary medicine, as in animals the predisposing constitution would remain in the animal, and the consumer would be as suspicious of the milk and beef of a cow that had been cured, as he would be of the diseased animal. On discussion of Dr. Birch's case of tetanus, Dr. Huidekoper cited several clinical cases in support of the theory of contagion of the disease. Drs. Glass and Kooker stated that they had not had succeeding cases in the same stables, and doubted its contagiosity. Dr. Landes reported that he had been experimenting for some time with cultures of discharges from the wounds in cases of tetanus. He had discovered a micro-organism, bacillus, which would not grow in the presence of oxygen; it grew at the temperature of the room; his experiments were not complete, but he believed he had found the cause of the disease. He would report at a later time. Dr. Lusson reported a case of a runaway horse which collided with a granite post six feet high and three feet square. The post, which probably weighed several tons, was knocked from its base and broken in two; the horse fractured its sternum and had to be destroyed. Dr. Hoskins reported a case which had been under his care from May 26th, 1888, until November 8th, 1890. It had from time to time shown symptoms, for a few days, resembling an attack of influenza. The autopsy showed a massive cyst in the lumbar region, surrounding diseased ovaries. It had never exhibited enlargement of the abdomen or symptoms of intestinal disturbance. Dr. Ridge recalled the similarity of the symptoms of this case with the one of mesenteric abscesses, which he had reported several years ago.

Dr. Zuill demanded that the charges against him be considered. Dr. Hoskins stated that no charges had been made, that his resolutions presented last month were in regard to the University of Pennsylvania, and were not charges. Dr. Zuill insisted that charges had been made. Dr. Hoskins then called Dr. Ridge to the chair and presented the following:

December 6th, 1890.

*To the Board of Trustees of the Keystone Veterinary Medical Association:*

I beg leave to request your opinion as to the legality of a member of this Association holding a position on the staff of the Veterinary Department of the University of Pennsylvania, so long as that institution assumes the present position of offering free services to all who may apply.

Respectfully submitted,

W. HORACE HOSKINS.

The communication was referred to the board of trustees.

Dr. Huidekoper requested that the board of trustees would not delay in acting upon Dr. Zuill's charges against him.

The president appointed Dr. Birch to act in the place of Dr. Harger on the board of trustees, when the charges against the University of Pennsylvania are brought before that board, and instructed the board to meet before the January meeting. Adjourned. C. H. MAGILL, *Secretary.*

